# Regulation of mitochondrial trifunctional protein modulates nonalcoholic fatty liver disease in mice

**DOI:** 10.1194/jlr.M080952

**Published:** 2018-03-26

**Authors:** Fatiha Nassir, Justin J. Arndt, Sarah A. Johnson, Jamal A. Ibdah

**Affiliations:** Departments of Medicine-Division of Gastroenterology and Hepatology,* University of Missouri, Columbia, MO; Nutrition and Exercise Physiology,† University of Missouri, Columbia, MO; Medical Pharmacology & Physiology,§ University of Missouri, Columbia, MO; United States Research Service,** Harry S. Truman Memorial Veterans’ Hospital, Columbia, MO

**Keywords:** sirtuin 3, acetylation, mitochondrial trifunctional protein, mitochondria

## Abstract

Mitochondrial trifunctional protein (MTP) plays a critical role in the oxidation of long-chain fatty acids. We previously reported that aging mice (>9 months old) heterozygous for an MTP defect (MTP^+/−^) develop nonalcoholic fatty liver disease (NAFLD). We tested whether a high-fat diet (HFD) accelerates NAFLD in young MTP^+/−^mice, and whether overexpression of the nicotinamide adenine dinucleotide (NAD^+^)-dependent deacetylase sirtuin 3 (SIRT3) deacetylates MTP and improves mitochondrial function and NAFLD. Three-month-old WT and MTP^+/−^ mice were fed HFD (60% cal fat) for 16 weeks and livers were assessed for fatty acid oxidation (FAO) and NAFLD. Compared with WT, MTP^+/−^ mice displayed reduced hepatic SIRT3 levels and reduced FAO, with increased hepatic steatosis and the inflammatory marker CD68. Hepatic overexpression of SIRT3 in HFD-fed MTP^+/−^ mice increased hepatic MTP protein levels at the posttranscriptional level. Immunoprecipitation of MTP from liver mitochondria followed by Western blot with acetyl-lysine antibody showed higher acetylation of MTP in MTP^+/−^ compared with WT mice. Overexpression of SIRT3 in MTP^+/−^ mice significantly reduced the acetylation of MTP compared with β-galactosidase controls, increased mitochondrial FAO, and reduced hepatic steatosis, CD68, and serum ALT levels. Taken together, our data indicate that deacetylation of MTP by SIRT3 improves mitochondrial function and rescues NAFLD in MTP^+/−^ mice.

Mitochondrial trifunctional protein (MTP) is a multienzyme complex composed of 4 α− and 4 β-subunits that catalyzes the last three steps in the β-oxidation of long-chain fatty acids. The α-subunit contains the activities of the long-chain enoyl-CoA hydratase and long-chain 3-hydroxyacyl-CoA dehydrogenase, and the β-subunit contains the activity of the long-chain thiolase (1). HADHA and HADHB are the genes encoding the α- and β-subunits, respectively. Human defects in MTP are recessively inherited and manifest as Reye-like syndrome and sudden infant death. Previously, we generated a knockout mouse model for the MTP α-subunit null allele causing complete MTP deficiency (2). Similar to the human deficiency, mice homozygous for the MTP defect develop hepatic steatosis and suffer sudden neonatal death (2). In subsequent work, we have demonstrated that aging (>9 months old) heterozygous (MTP^+/−^) mice develop nonalcoholic fatty liver disease (NAFLD) on chow diet concomitant with reduced mitochondrial fatty acid oxidation (FAO) (3–5).

NAFLD is a spectrum that includes hepatic steatosis, nonalcoholic steatohepatitis (NASH), and may progress to cirrhosis and hepatocellular carcinoma (6–8). NAFLD is strongly associated with obesity, insulin resistance, and enhanced risk of cardiovascular disease (9, 10). Currently, NAFLD is the most prevalent form of liver disease in both children and adults, and is the primary cause of liver failure. Apart from life style modifications such as calorie restriction and exercise, no effective pharmacological treatment currently exists to treat NAFLD (7, 11). This is mainly due to the lack of clear understanding of the underlying pathophysiology of the disease.

Strong evidence supports a crucial role for the mitochondria in the pathophysiology of NAFLD (3, 5, 10, 12, 13). Mitochondrial dysfunction is a common finding in NAFLD patients and in animal models of NAFLD where ultrastructural abnormalities of liver mitochondria, reduced FAO, impaired hepatic ATP synthesis, reduced respiration, and increased oxidative stress have been observed (2, 5, 12–17).

Sirtuins (SIRTs) are a family of seven proteins (SIRT1–SIRT7), three of which are localized in the mitochondria (SIRT3–5). SIRT3 is a nicotinamide adenine dinucleotide (NAD^+^)-dependent deacetylase that regulates the function of many mitochondrial proteins and may play a protective role against NAFLD (18–20). SIRT3-deficient mice fed a chronic high-fat diet (HFD) develop accelerated obesity, insulin resistance, and steatohepatitis compared with WT mice (21). Among the mitochondrial sirtuins, SIRT3 possesses the most robust deacetylase activity (22). SIRT3-deficient mice show increased hyperacetylation of mitochondrial proteins and reduced FAO compared with WT mice (23, 24). Label-free quantitative mass spectrometry analysis of the lysine acetylome from SIRT3-deficient mice identified the MTP α-subunit as a highly acetylated protein (24). It is not known whether SIRT3 gain-of-function rescues NAFLD, particularly in our mouse model of mitochondrial dysfunction.

Herein, we propose that regulation of MTP modulates NAFLD. MTP plays a central role in FAO and hence, factors that increase its activity are likely to rescue NAFLD by improving FAO. However, little is known about MTP regulation. In this study, we tested whether SIRT3 regulates MTP via deacetylation and whether deacetylation of MTP is an underlying mechanism in the rescue of NAFLD in our mouse model. We utilized an HFD in young MTP^+/−^ mice to accelerate development of NAFLD. In addition, we overexpressed SIRT3 in the liver of these mice to determine whether deacetylation of MTP is an underlying mechanism in the regulation of MTP and NAFLD.

## METHODS

### Animals, diet, and adenoviral injection

The animal protocol was approved by the institutional Animal Care and Use Committee at the University of Missouri-Columbia. Male mice on a C57BL/6 background were used in these studies (2). WT and MTP^+/−^ mice genotype was determined by PCR using primers that differentiate the mutant from the WT allele, as described previously (2). Mice were housed in temperature-controlled rooms (21°C) with 06.00–18.00 h light: 18.00–06.00 h dark cycles that were maintained throughout the experimental period. Mice were given standard (chow) diet (Formulab 5008, Purina Mills, St. Louis, MO) or HFD (D12492, Research Diets, Inc., 60% cal fat). For HFD experiments, animals (three months old) were given HFD for 16 weeks after which they were euthanized. For SIRT3 studies, three-month-old WT and MTP^+/−^ mice were given HFD for 16 weeks after which adenoviral expression of β-galactosidase (β-gal) (control) or SIRT3 (abm, Richmond, BC) was performed (9× 10^9^ virus particles/g body weight, diluted in saline) via tail vein injection of the adenovirus and the animals were studied 3 days postinjection. The virus was amplified and purified using Add-N-Pure Adenovirus Purification Kit (abm, Richmond, BC) as described by the manufacturer. All mice were fasted overnight, anesthetized with sodium pentobarbital (50 mg/kg), and blood and tissues were collected. Livers and other tissues were frozen in liquid nitrogen for biochemical analysis and Western blot analysis or stored in RNA-later solution for gene expression studies. Tissue samples were also fixed using 10% neutral buffered formalin for histological analysis, or in Tissue-Tec O.C.T. to prepare frozen sections.

### Isolation of mitochondria and measurement of FAO

Mitochondria were prepared from liver samples as described previously (25) and according to modified method of Koves et al. (26). The oxidation of fatty acids in the liver and isolated mitochondria was measured using ^14^C-Palmitate (American Radiochemical) in fresh tissues and isolated liver mitochondria as described previously (27). The ^14^CO2 resulting from complete combustion of ^14^C-Palmitate was counted using a liquid scintillation counter.

### Western blotting

Homogenates from liver or mitochondria were separated on 4-20% SDS-PAGE gels, transferred to nitrocellulose membranes, and probed with specific antibodies to MTP, Acetyl-Lysine, SIRT1 and SIRT3 (Cell Signaling), SIRT5 (Abcam), SIRT4, SIRT7, tubulin (Santa Cruz), SIRT6 (MyBioSource, San Diego, CA). Proteins were visualized with the molecular imager ChemiDOC XRS^+^ and the intensities of the bands were quantified using Quantity One Analysis Software (Bio-Rad).

### RNA extraction and RT PCR

Total RNA was extracted from liver samples using RNeasy Mini Kit (Qiagen) and subjected to cDNA Reverse Transcription (Applied Biosystems) and RT quantitative PCR using Power SYBR Green PCR Mix on StepOnePlus Real-Time PCR System (Applied Biosystems). The data were analyzed using the StepOne software using specific real-time PCR primers. The real time primers for HADHA were purchased from Bio-Rad.

### Acetylation of MTPα

Mitochondria were isolated from liver tissue as described above and mitochondrial pellets were resuspended in lysis buffer containing protease inhibitors. MTPα was immunoprecipitated from mitochondrial lysate using anti-MTPα antibody crosslinked to protein A/G agarose beads. The crosslinking of the antibody to protein A/G agarose beads and the immunoprecipitation of MTPα was conducted as described in the Pierce Crosslink Immunoprecipitation Kit (Thermo Scientific). Equal amounts of immunoprecipitated proteins were separated on SDS-PAGE and transferred to nitrocellulose membrane, which was then probed either with anti-MTPα antibody to determine the mitochondrial level of MTPα or with anti-acetyl lysine to determine its acetylation level.

### Hepatic lipid content and histology

Hepatic lipids were extracted using chloroform-methanol (2:1, v/v) and hepatic triglyceride content was analyzed using a triglyceride assay kit (Wako diagnostics, Moutain View, CA). Oil Red O staining for neutral lipids was determined using liver frozen sections. Staining of liver sections for H&E and CD68 was performed using formalin fixed liver sections.

### Statistics

Statistical significance was determined with Student’s *t*-test. Data are expressed as mean ± SE; a *P* value < 0.05 was considered statistically significant.

## RESULTS

### HFD accelerates NAFLD in MTP^+/−^ mice

**Fig. 1.**
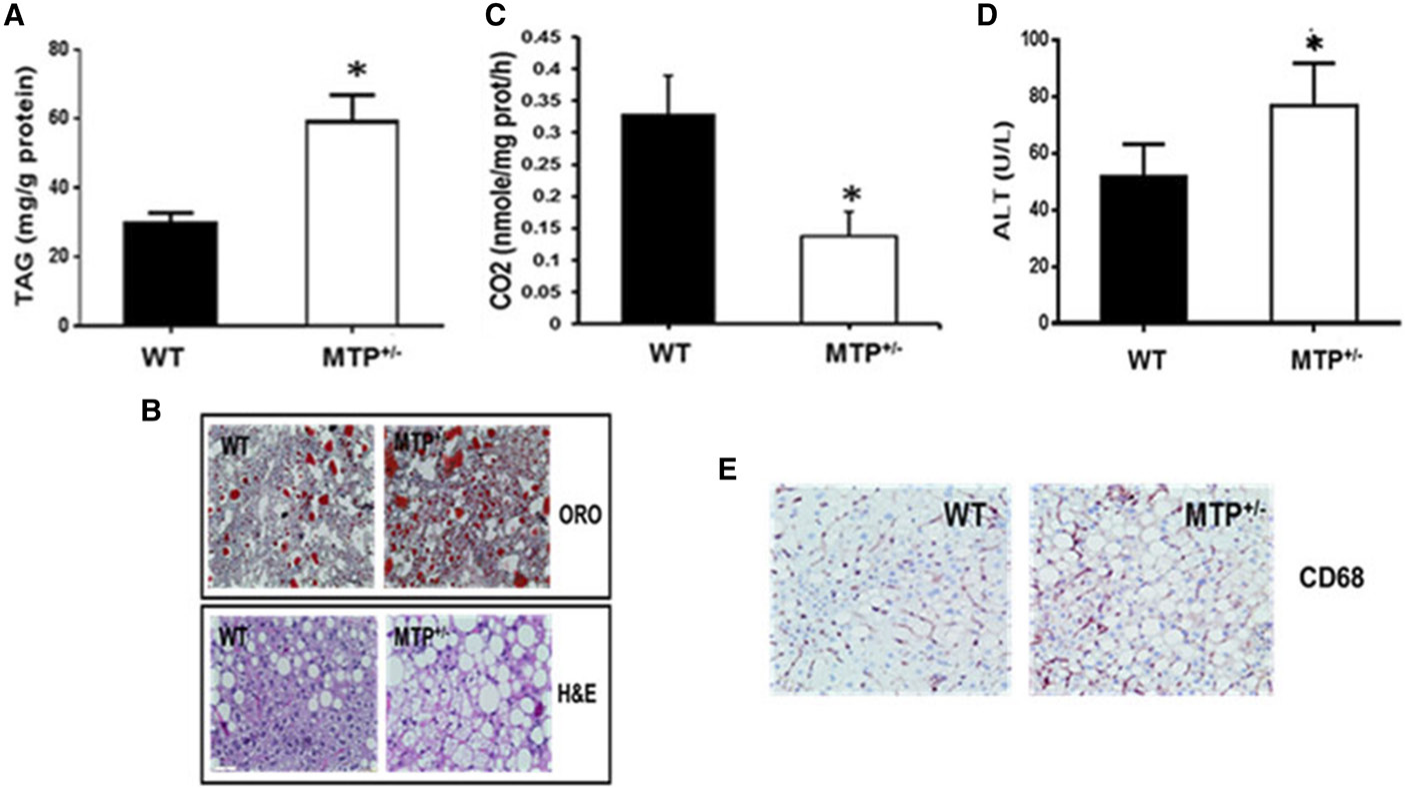
HFD accelerates hepatic steatosis and liver inflammation in young MTP^+/−^ mice. A: Hepatic triglyceride (TAG). B: Oil Red O (ORO) and H&E staining of liver sections. C: Fatty acid oxidation in mitochondria from WT and MTP^+/−^ mice. D: Serum ALT. E: CD68 staining of liver sections. Data are mean ± SE; n = 6; *P < 0.05 using Student’s *t*-test.

All mice gained ∼10 g in body weight on HFD but no differences in weight gain, weekly food intake, or body and liver weight were observed at the end of the 16 week HFD between the WT and MTP^+/−^ mice (**Table 1**). MTP^+/−^ mice on HFD had 2-fold increase in hepatic lipid accumulation compared with WT mice (**Fig. 1A**). This was confirmed by Oil Red O staining for neutral lipids and H&E staining (Fig. 1B) of liver sections. The increase in hepatic triglyceride in MTP^+/−^ mice was associated with >50% reduction in FAO compared with WT (Fig. 1C). MTP^+/−^ mice also displayed increased levels of serum ALT (Fig. 1D) and CD68 (Fig. 1E) compared with WT.

**TABLE 1.** Effect of HFD on food intake, weight gain, and body and liver weight in WT and MTP^+/−^ mice

Mice	WT	MTP^+/−^
Average weekly food intake (g)	15.7 ± 0.02	16.8 ± 0.03
Average weight gain (g)	12.0 ± 0.9	11.1 ± 1.0
Body weight (g)	43.5 ± 1.4	41.2 ± 0.8
Liver weight (g)	2.0 ± 0.3	1.8 ± 0.3

Data are expressed as means ± SEM of n = 10, no statistical difference, *P* > 0.5 using *t*-test.

### SIRT3 levels are reduced in MTP^+/−^ mice

**Fig. 2.**
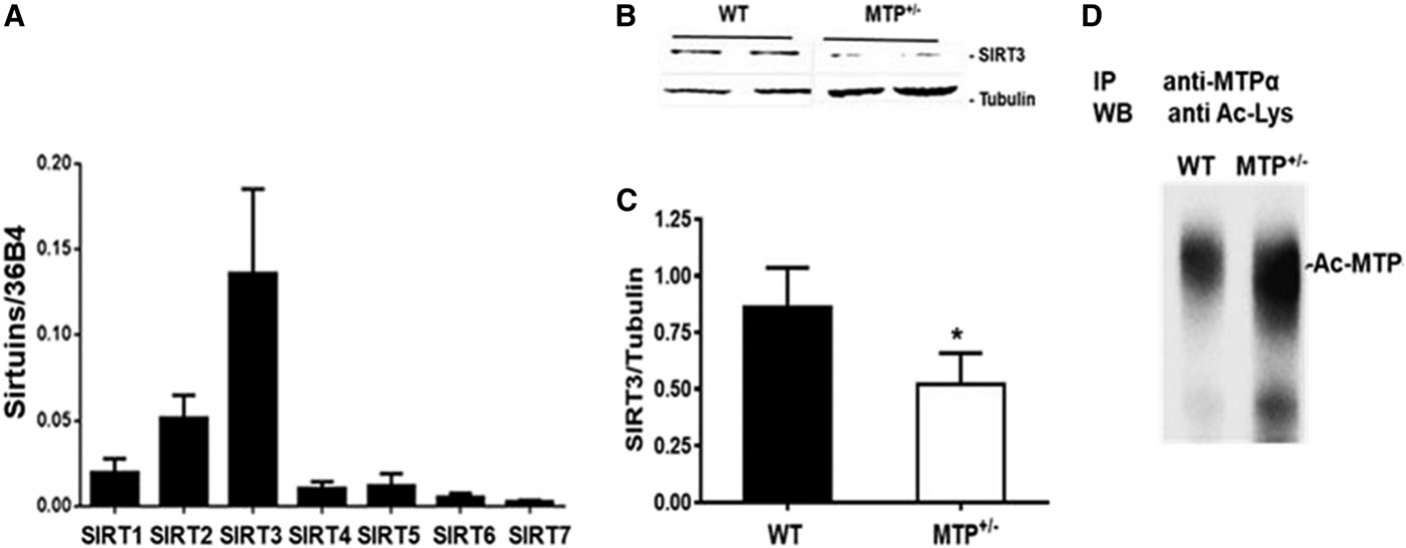
SIRT3 levels are reduced in MTP^+/−^ mice. A: Hepatic expression of the different sirtuins in WT mice. B: Hepatic levels of SIRT3 in young WT and MTP^+/−^ mice fed HFD. C: Quantification of hepatic levels of SIRT3. D: HADHA was immunoprecipitated from mitochondrial lysate from WT and MTP^+/−^ mice and equal amount of immunoprecipitated proteins were probed for Ac-Lys. Data are mean ± SE; n = 3–6.

We examined the abundance of the different sirtuins in WT livers and found that SIRT3 is the highly expressed sirtuin in this organ (**Fig. 2A**). Importantly, hepatic SIRT3 levels were reduced in MTP^+/−^ mice compared with WT (Fig. 2B, C), and this was concomitant with higher acetylation of MTP in the livers of MTP^+/−^ compared with WT mice (Fig. 2 D).

### Overexpression of SIRT3 reduced hepatic triglyceride in MTP^+/−^ mice

**Fig. 3.**
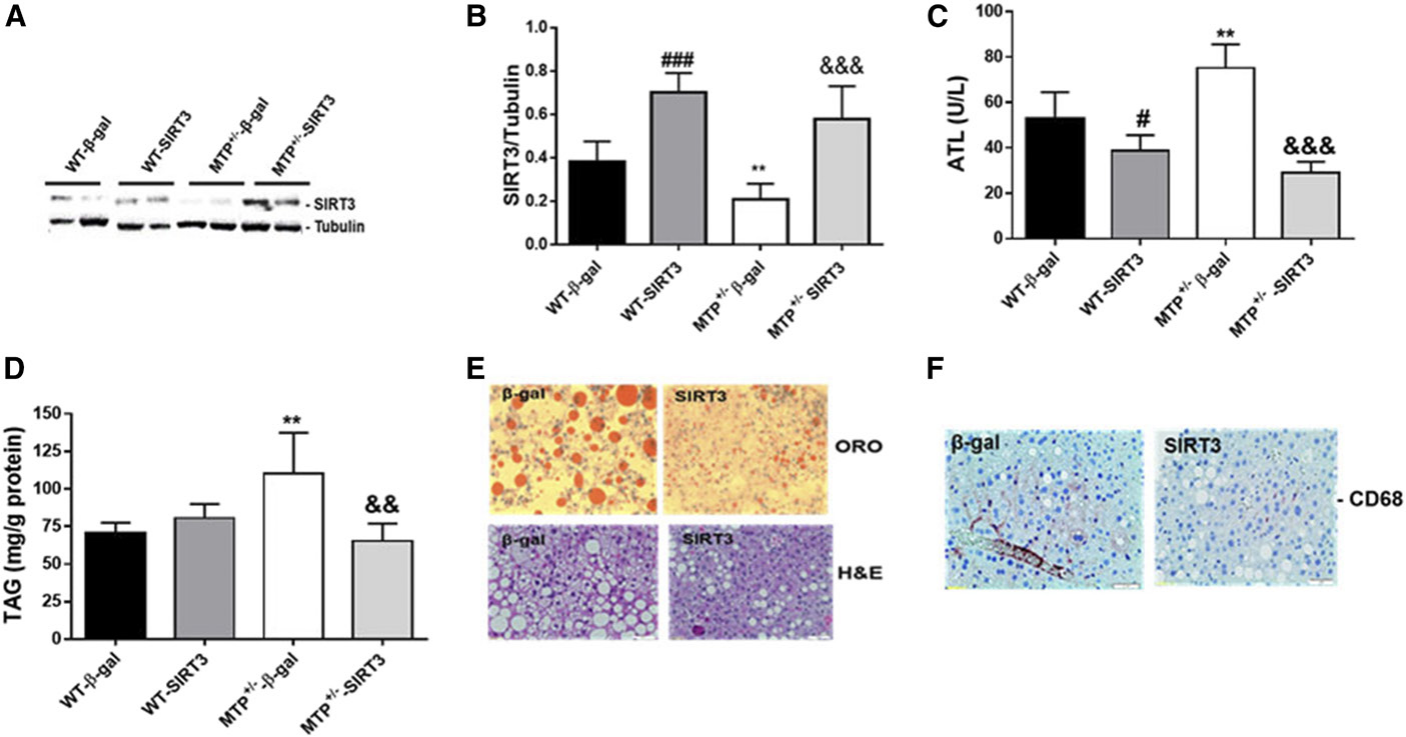
Overexpression SIRT3 improves NAFLD in MTP^+/−^ mice. A: Overexpression of SIRT3 in WT and MTP^+/−^ mice. B: Quantification of hepatic SIRT3 levels in WT type and MTP^+/−^ mice injected with β-gal or SIRT3 adenovirus. C: ALT levels. D: Hepatic triglyceride (TAG). E: Oil Red O (ORO) and H&E staining of liver sections from MTP^+/−^ mice injected with β-gal or SIRT3 adenovirus. F: Staining of liver sections from MTP^+/−^ mice injected with β-gal or SIRT3 adenovirus. Data are mean ± SE; n = 3–6. #, difference between WT β-gal and WT SIRT3; *, difference between WT-β-gal and MTP^+/−^-β-gal; &, difference between MTP^+/−^-β-gal and MTP^+/−^- SIRT3.

We used mouse SIRT3 adenovirus to specifically overexpress SIRT3 in the liver of WT and MTP^+/−^ mice. SIRT3 overexpression increased hepatic SIRT3 levels in both WT and MTP^+/−^ livers (**Fig. 3A, B**) and reduced ALT levels minimally in WT and dramatically in MTP^+/−^ compared with β-gal-injected mice (Fig. 3C). Hepatic triglyceride levels were higher in MTP^+/−^ compared with WT β-gal mice (Fig. 3D). Overexpression of SIRT3 reduced triglyceride levels in MTP^+/−^ but not in WT livers (Fig. 3D). Oil Red O and H&E staining confirmed the reduction in hepatic triglyceride in MTP^+/−^ mice upon SIRT3 overexpression (Fig. 3E). SIRT3 overexpression also reduced the inflammatory marker CD68 in MTP^+/−^ livers (Fig. 3F).

### SIRT3 deacetylates MTP and restores its levels in MTP^+/−^mice

**Fig. 4.**
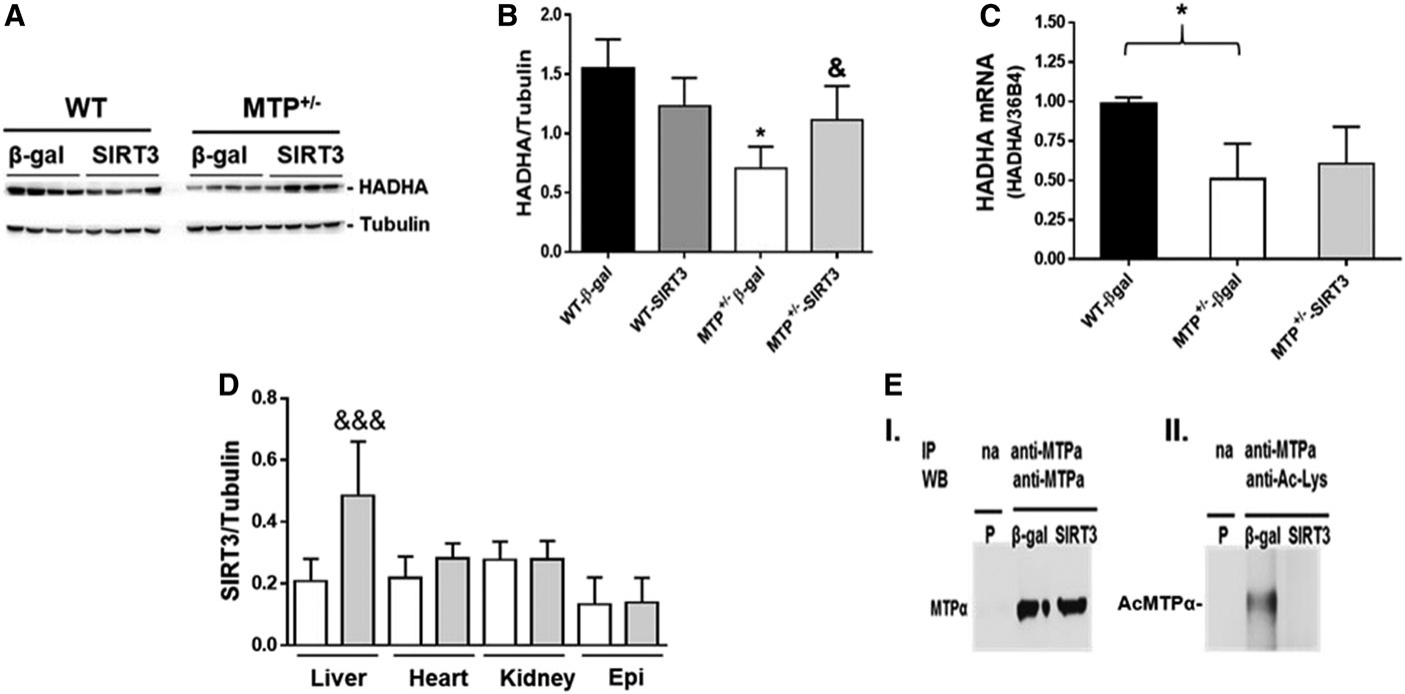
SIRT3 upregulates MTP in MTP^+/−^ mice. A: Hepatic levels of HADHA in WT and MTP^+/−^ mice expressing β-gal and SIRT3. B: Quantification of HADHA levels. C: Hepatic HADHA mRNA level. D: Specific overexpression of SIRT3 in the liver; white bars, MTP^+/−^-β-gal; gray bars, MTP^+/−^-SIRT3. E: Immunoprecipitation (IP) of HADHA from liver mitochondrial lysates from mice injected with β-gal or SIRT3 adenovirus followed by Western blot (WB) for HADHA (I) or for acetyl-lysine to detect acetylated MTPα (Ac-MTPα) (II). P, pool of mitochondrial lysate; na: no antibody. Data are mean ± SE; n = 3–4. *, difference between WT-β-gal and MTP^+/−^-β-gal; &, difference between MTP^+/−^-β-gal and MTP^+/−^-SIRT3.

We examined hepatic MTP levels in WT and MTP^+/−^ mice injected with control β-gal or SIRT3 adenovirus. As expected, the MTP protein (**Fig. 4A, B**) and mRNA levels (Fig. 4C) were ∼50% lower in the MTP^+/−^ compared with WT β-gal injected mice. SIRT3 overexpression restored hepatic MTP protein levels to WT levels (Fig. 4A, B) without an increase in MTP gene expression (Fig. 4C), suggesting that SIRT3 regulates MTP levels at the posttranscriptional level. The overexpression of SIRT3 in MTP^+/−^ mice was specific to the liver, as no change was observed in other tissues (Fig. 4D). SIRT3 overexpression also had no effect on body and liver weight (**Table 2**). To determine whether SIRT3 deacetylates MTPα, we subjected liver mitochondrial lysates from MTP^+/−^ mice injected with control or SIRT3 adenovirus to immunoprecipitation with anti- MTPα antibody. Equal amounts of MTPα immunoprecipitates were subjected to Western blot with either MTPα or acetyl lysine (Ac-Lys). As expected, shows equal amounts of MTPα protein immunoprecipitated from both β-gal and SIRT3 overexpressing mice. However, Western blot with Ac-Lys antibody shows significantly lower acetylation of MTPα in the SIRT3 compared with β-gal controls (Fig. 4E-II). These data document in vivo deacetylation of MTP by SIRT3.

**TABLE 2.** Effect of SIRT3 injection on body and liver weight

Mice	MTP^+/−^-β-gal	MTP^+/−^-SIRT3
Body weight (g)	41.9 ± 1.4	42.7 ± 1.2
Liver weight (g)	1.76 ± 0.2	1.78 ± 0.1

Data are expressed as means ± SEM of n = 10, no statistical difference, *P* > 0.5 using *t*-test.

### SIRT3 overexpression improves mitochondrial function in MTP^+/−^mice

**Fig. 5.**
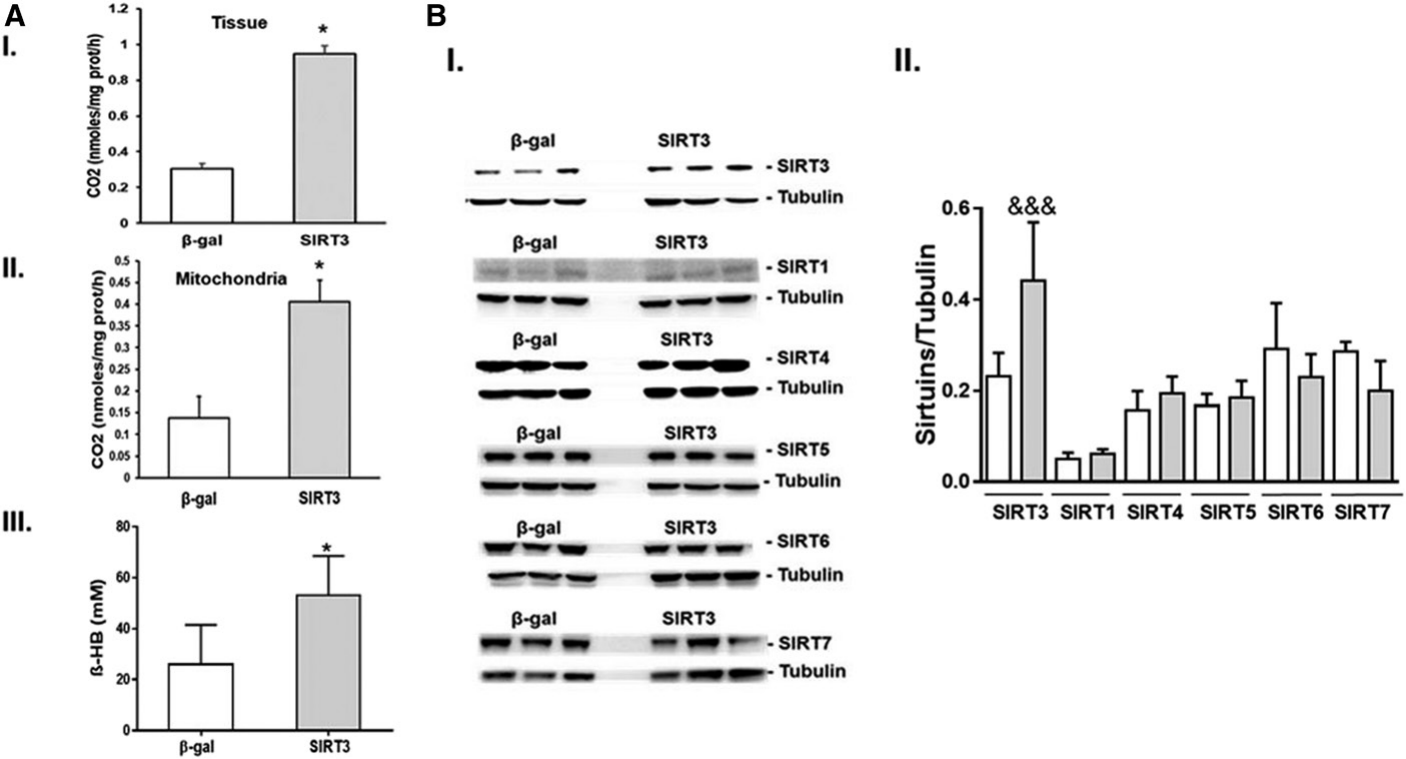
Adenoviral overexpression of SIRT3 in vivo restores FAO in young MTP^+/−^ mice on HFD. A: Complete oxidation of ^14^C-radiolabeled palmitate to CO2 in liver tissue (I) and isolated mitochondria (II), and serum levels of β-Hydroxybutyrate (β -HB) (III). B: Hepatic levels of the different sirtuins in the liver from mice injected with β-gal or SIRT3 adenovirus (I) and quantifiquation (II). Data are mean ± SE; n = 5.

Mitochondrial function was evaluated in SIRT3 and β-gal injected MTP^+/−^ mice. FAO was increased in both liver tissue (**Fig. 5A-I**) and in isolated mitochondria (Fig. 5A-II) from mice overexpressing SIRT3 compared with β-gal controls. Blood levels of β-hydroxybutyrate were also increased in SIRT3 overexpressing livers as compared with β-gal controls (Fig. 5A-III), consistent with increased FAO. The improvement in hepatic mitochondrial function in mice overexpressing SIRT3 were due to the specific increase in the hepatic level of SIRT3 without significant changes in the protein levels of other sirtuins (Fig. 5B-I,II).

## DISCUSSION

MTP is a multienzyme protein complex that plays a major role in mitochondrial FAO as it carries three of the four enzymes required for long-chain FAO. We previously reported that MTP deficiency in mice results in hepatic steatosis, cardiomyopathy, and neonatal sudden death (2), whereas aging heterozygous MTP^+/−^ mice develop hepatic steatosis and insulin resistance (3, 5). In the current study, we show that HFD accelerates NAFLD in susceptible young MTP^+/−^ mice, which was associated with highly acetylated MTP α-subunit and reduced FAO. We have shown that hepatic overexpression of SIRT3 reduces MTP acetylation levels, restores FAO, and improves NAFLD in mice.

MTP catalyzes three consecutive steps in the mitochondrial β-oxidation of long-chain acyl-CoA esters: 2-enoyl-CoA hydratase, 3-hydroxyacyl-CoA dehydrogenase, and 3- ketoacyl-CoA thiolase. MTP enzymatic deficiencies are important causes of human disease. Formation of a protein complex between α− and β-subunits is important for MTP stability. This is supported by the observation that individual α- and β-subunits are unstable when expressed heterologously (28). Genes for the α- and β-subunits are adjacent to each other in a head-to-head configuration on chromosome 2p23, and are transcribed from the same bidirectional promoter region (29); hence, expression of the two subunits may be coordinated. A unique feature is the association between MTP defects and acute fatty liver of pregnancy (30). Mothers carrying fetuses with 3-hydroxyacyl-CoA dehydrogenase deficiency develop a life-threatening condition of acute fatty liver of pregnancy, highlighting the important role of MTP in development of fatty liver disease. Given the key role of MTP in FAO and its significance to human disease, understanding of MTP regulation is of paramount importance.

In the present study, we document that HFD accelerates NAFLD in susceptible young MTP^+/−^ mice, which was associated with reduced SIRT3 levels, highly acetylated MTP α-subunit, and reduced FAO. Reduced SIRT3 levels were also observed in aging MTP^+/−^ mice (data not shown). Interestingly, hepatic SIRT3 was also reported to be reduced in mouse models of NAFLD and in NASH patients compared with control subjects (23, 31) suggesting a significant role for SIRT3 in NAFLD. We show that hepatic overexpression of SIRT3 improved NAFLD in the MTP^+/−^ mouse model but had no effect on lipid accumulation in WT mice. This is consistent with reports that overexpression of SIRT3 in C57B6 mice liver in vivo did not protect against lipid accumulation induced by HFD, whereas SIRT3 deficient mice fed an HFD show accelerated obesity, insulin resistance, hyperlipidemia, and steatohepatitis compared with WT mice (21, 32). The observed beneficial effect of SIRT3 overexpression in the MTP ^+/−^ mouse model is intriguing and may suggest that MTP ^+/−^ mice benefited the most from SIRT3 overexpression due to the higher MTP acetylation status and lower hepatic SIRT3 levels in these mice. The reduced SIRT3 level in this mouse model warrants additional studies to explore the underlying mechanisms. It is possible that knockdown of the MTP gene downregulates SIRT3 levels. We have documented in an earlier study that *MTPα^+/−^* mice display enhanced extramitochondrial FAO with higher reactive oxygen species generation compared with *WT* mice (3). Several recent studies have reported attenuated SIRT3 expression in response to enhanced reactive oxygen species generation (33, 34).

Our data document that overexpression of SIRT3 in the mouse liver reduces the acetylation of MTP and increases hepatic MTP protein levels without alterations in HADHA gene expression, suggesting that SIRT3 regulates MTP at the posttranscriptional level. The reduced MTP acetylation and the improvement in the oxidation of fatty acids in the mitochondria of MTP^+/−^ mice upon SIRT3 overexpression was independent from changes in the hepatic levels of other sirtuins, indicating a direct role of SIRT3 in the regulation of MTP. Thus, SIRT3 may enhance FAO and mitochondrial function through deacetylation and increased stability of MTP. The mechanism by which lysine acetylation causes unstable MTP complex is unknown and requires future studies to investigate how SIRT3 specifically enhances MTP stability.

In summary, we show that SIRT3 regulates hepatic levels of MTP by modulating its acetylation status, which in turn leads to regulation of mitochondrial FAO, impacting development and rescue of NAFLD.
